# RNA-based drugs and regulation: Toward a necessary evolution of the definitions issued from the European union legislation

**DOI:** 10.3389/fmed.2022.1012497

**Published:** 2022-10-17

**Authors:** Mathieu Guerriaud, Evelyne Kohli

**Affiliations:** ^1^CREDIMI Laboratory EA 7532 and Laboratory of Excellence LipSTIC ANR-11-LABX-0021, Faculty of Health Sciences (Pharmacy), University of Burgundy, Dijon, France; ^2^UMR INSERM/uB/AGROSUP 1231, Team 3 HSP-Pathies, Labelled Ligue Nationale Contre le Cancer and Laboratory of Excellence LipSTIC ANR-11-LABX-0021, Faculty of Health Sciences (Pharmacy), University of Burgundy, Dijon, France

**Keywords:** RNA-based drugs, mRNA vaccines, gene therapy medicinal products (GTMP), advanced therapy medicinal products (ATMP), biological medicinal products, EMA, FDA, ICH

## Abstract

Many RNA-based drugs, both vaccines and non-vaccines, are under development or even approved. They include coding mRNAs and non-coding (nc) RNAs among them antisense oligonucleotides (ASOs), small interfering RNAs (siRNAs), micro-RNAs (miRNAs), small activating RNAs (saRNAs), RNA aptamers and RNA guides. According to the European Union (EU) legislation, these products can be currently categorized into different regulatory statuses, depending, for vaccines, on their target (infectious disease or not) and, for other drugs, on how they are obtained (chemically or biologically). This classification is fundamental to the type of marketing authorization (MA), and therefore to the controls to be performed, from preclinical stages through clinical trials to pharmacovigilance, to meet the safety requirements for patients. However, the current rules raise several problems, in particular the risk, because technology is evolving, to have similar RNA drugs being covered by very different legal statuses and the lack of international harmonization. The objectives of this study are (i) to review how RNA medicinal products are currently legally categorized in the EU and especially whether they fall under the status of gene therapy medicinal products (GTMP), a regulatory status belonging to advanced therapy medicinal products (ATMP), (ii) to discuss the issues generated by this classification, with a focus on the heterogeneity of statuses of these products, the differences with the American and ICH definitions and the potential impact on the safety requirements.

## Introduction

While the first mRNA vaccines against an infectious disease have reached the market (1, 2), many other medicinal products with RNA as an active substance (see Table 1), either vaccines against non-infectious diseases or non-vaccine drugs, are under development (3–18) or even approved (19–25).

**TABLE 1:** Examples of RNA-based drugs currently or formerly on the market or under development.

Type of RNA	Subcategory (if applicable)	Drugs in the EU (green: approved/orange: withdrawn/yellow: under development)	Therapeutic indication	Regulatory status italics: probable status for MP under development)
mRNA	mRNA	UX053 NCT04990388 (3)	Glycogen Storage Disease Type III (GSD III)	*GTMP*
	
	mRNA-based vaccines against an infectious disease	Comirnaty^®^ tozinameran (1) Spikevax^®^ elasomeran (2)	active immunization to prevent COVID-19 caused by SARS-CoV-2 virus	Vaccine
	
	mRNA-based vaccines for the treatment of cancer: direct injection of mRNA	IVAC MUTANOME^®^ Phase I Clinical Trial NCT02035956 (4, 5)	advanced melanoma	*GTMP*
	
	mRNA-based vaccines for the treatment of cancer: mRNA cell therapies	autologous mature DCs co-electroporated with *in vitro* transcribed autologous renal cell RNA and CD40L RNA NCT00678119(6)	metastatic clear cell renal cell carcinoma	*sCTMP*
	
	mRNA based CAR-T cells produced *ex vivo*	Sparkcures Descartes-08 CAR-T cells NCT04816526 (7, 8)	high-risk multiple myeloma	*sCTMP*
	
	mRNA based CAR-T cells produced *in vivo*	Preclinical step. Proof of concept murine model (9)	cardiac fibrosis	*GTMP*

Antisense Oligonucleotides (ASOs)		Spinraza^®^ nusinersen (19)	5q spinal muscular atrophy	“chemical”
	
		Tegsedi^®^ inotersen (20)	stage 1 or stage 2 polyneuropathy in adult patients with hereditary transthyretin amyloidosis	“chemical”
	
		Waylivra^®^ volanesorsen (21)	[…] genetically confirmed familial chylomicronemia syndrome (FCS) […]	“chemical”
	
		*Withdrawn: Kyndrisa*^®^ *drisapersen* (69)	*was expected to be used for the treatment of Duchenne muscular dystrophy*	“chemical”

RNA interference (RNAi)	small interfering RNAs (siRNAs)	Onpattro^®^ patisiran (22)	hereditary transthyretin-mediated amyloidosis (hATTR) in adults with stage 1 or stage 2 polyneuropathy	“chemical”
	
		Givlaari^®^ givosiran (23)	acute hepatic porphyria (AHP) in adults and adolescents aged 12 years and older	“chemical”
	
		Oxlumo^®^ lumasiran (24)	primary hyperoxaluria type 1 (PH1) in all age groups	“chemical”
	
		Leqvio^®^ inclisiran (25)	primary hypercholesterolemia or mixed dyslipidemia in adults	“chemical”
	
	micro-RNAs (miRNAs)	Remlarsen (MRG 201) NCT03601052 (10, 11)	Keloid scar (target miR-29)	*“chemical”*
	
		Lademirsen (SAR339375/RG 012) NCT02855268 (12)	Alport syndrome (target miR-21)	*“chemical”*

RNA activation (RNAa)	small activating RNA (saRNA)	MTL-CEBPA (13) NCT05097911 (14) NCT04710641 (15)	advanced hepatocellular carcinoma (target CEBPA)	*“chemical”*

RNA aptamers		*Withdrawn: Macugen*^®^ *pegaptanib* (70)	*was indicated for the treatment of neovascular (wet) age-related macular degeneration (AMD) in adults.*	“chemical”

RNA Guide/Direct genome editing*		NTLA-2001 (mRNA for Cas9 combined with a single short guide RNA) NCT04601051 (16, 17)	hereditary transthyretin amyloidosis	*GTMP*
	
		Nanoparticles formed by a cationic 4-armed polymer containing the gene editing components (CRISPR/Cas9 and the single guide RNAs) (18)	treatment of recessive dystrophic epidermolysis bullosa	*Biological MP*

*Drug status is determined by the associated nuclease.

Currently, according to the EU legislation, which was written before most RNA products were developed, these medicinal products fall under several different statuses, i.e., vaccines, advanced therapy medicinal products (ATMP), simple biological medicinal products or “simple chemical medicinal products”, depending, for vaccines, on their targets, infectious *vs*. non-infectious diseases and for non-vaccine drugs, on the type of RNA substance and its production (26–28). However, things are becoming more and more complicated as the current evolution of technology in this field makes it possible to produce similar RNA-based drugs using different approaches.

Few regulatory studies are available to assist in the regulatory categorization of RNA-based medicinal products. They concern mRNA drugs, especially the classification of mRNA-based vaccines against infectious and non-infectious diseases (29) and the regulatory framework of mRNA-based therapeutics (30, 31). To our knowledge, no publication is available for other RNA-based drugs.

The objectives of this study are (i) to review how RNA medicinal products are currently legally categorized in the EU and especially whether they fall under the status of gene therapy medicinal products (GTMP), a regulatory status belonging to advanced therapy medicinal products (ATMP), (ii) to discuss the issues generated by this classification, with a focus on the heterogeneity of statuses of RNA-based medicinal products, the differences with the American and ICH definitions and the potential impact on the security requirements.

## The legal statuses of RNA-based medicinal products in the European union

There are currently several products containing RNA on the market, and many are under development, among them mRNA, antisense oligonucleotides (ASOs), small interfering RNAs (siRNAs), small activating RNAs (saRNAs), micro-RNAs (mi-RNAs), RNA aptamers and RNA guides. These medicinal products do not have the same legal status. First, we will remind the rules of classification in these statuses according to the EU legislation, then, we will analyze the statuses of approved or under development RNA-based medicinal products inside the EU.

### The possible legal statuses for RNA-based drugs or candidate drugs

**FIGURE 1:**
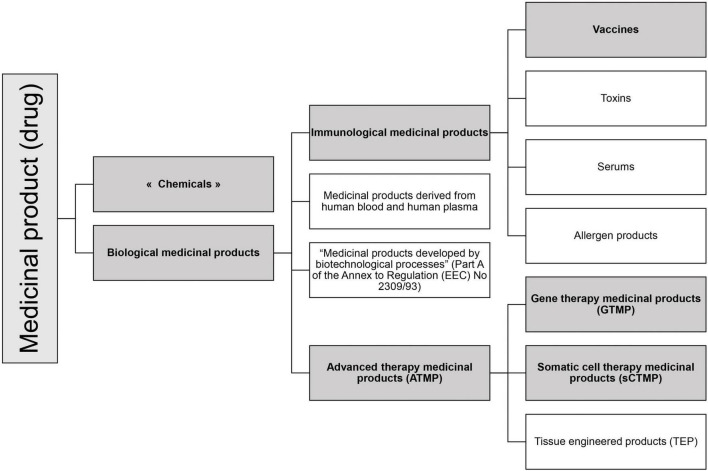
Medicinal product statuses according to EU legislation. In gray are represented the statuses in which RNA-based drugs can be categorized.

According to the EU regulation, the RNA-based drugs can be classified in various specific medicinal product statuses (see Figure 1).

Medicinal products are defined in the consolidated directive 2001/83/EC (27). They include chemicals and biological products.

#### Chemicals

Although there is no legal definition of a “chemical medicinal product”, the basis of the EU drug regulation concerns chemical drugs. Over time, new statuses have emerged to implement additional safety requirements, such as biological medicinal products status.

#### Biological medicinal products

According to the Consolidated Directive 2001/83/EC, Annex I, Part I, §3.2.1.1.b) (27):

“*A biological medicinal product is a product, the active substance of which is a biological substance.*”

And a biological substance is defined as “*a substance that is produced by or extracted from a biological source and that needs for its characterization and the determination of its quality a combination of physico-chemical-biological testing, together with the production process and its control.*”

The regulation mentioned four subcategories of biological medicinal products (Figure 1) of which two relate to RNA drugs: Immunological medicinal products including vaccines, and Advanced Therapy Medicinal Products.

##### Immunological medicinal products and vaccines

According to the consolidated Directive 2001/83/EC cited above (27), Article 1, §4, an immunological medicinal product is defined as “*Any medicinal product consisting of vaccines, toxins, serums or allergen products*.” As for vaccines, they are defined here as “*agents used to produce active immunity, such as cholera vaccine, BCG, polio vaccines, smallpox vaccine*.”

The European pharmacopoeia, for its part, defines them as: “*preparations containing antigens capable of inducing a specific and active immunity in man against an infecting agent or the toxin or antigen elaborated by it*” (32).

##### Advanced therapy medicinal products

Advanced therapy medicinal products (ATMP) are defined in Article 2 of Regulation (EC) No 1394/2007 as any of the following medicinal products for human use (28):

- a gene therapy medicinal product (GTMP),
- a somatic cell therapy medicinal product (sCTMP),
- a tissue engineered product (TEP).

Among these three statuses, GTMP is the most frequently used status for RNA-based drugs, but sCTMP also represents a possible status.

###### Gene therapy medicinal products

Gene therapy medicinal products (GTMPs) have been defined in the Annex I, Part IV, §2.1 of the Directive 2001/83/EC consolidated (27).

This definition is complex and involves several criteria (26):

1) the drug must belong to the status of a biological medicinal product. The active substance must therefore be a biological substance,
2) the drug must contain or consist of a nucleic acid, in other words, DNA or RNA, and this nucleic acid must be recombinant,
3) the sequence must be administered to regulate, repair, replace, add, or delete a genetic sequence,
4) the action must be therapeutic, prophylactic, or diagnostic and must be directly dependent on the nucleic acid used.

It should be noted that vaccines against infectious diseases are not included in GTMPs: *“Gene therapy medicinal products shall not include vaccines against infectious diseases.”*

###### Somatic cell therapy medicinal products

Somatic cell therapy medicinal products (sCTMP) are also defined in the Annex I, Part IV, §2.1 of the Directive 2001/83/EC consolidated.

1) As for GTMP, this definition is complex and involves several criteria (26): the drug must belong to the status of a biological medicinal product,
2) it must contain a cell or a tissue, both terms having definitions of their own,
3) these cells (or tissues) must either be substantially manipulated or not be intended to be used for the same essential function(s) in the recipient and the donor. In the first case, the concept of “substantial manipulation” is particularly important and examples are cited: “*Examples of substantial manipulations include cell expansion (culture), genetic modification of cells, differentiation/activation with growth factors*” (26).

These different regulatory statuses of drugs correspond to as many different controls. Biological drugs require specific controls compared to “chemical” drugs, as their size and inherent variability require the use of advanced analytical methods (e.g., peptide mapping, mass spectrometry…) to study their physicochemical and functional properties, such as molecular structure, protein modifications and biological activity (33, 34). In addition, their biological nature makes them susceptible to contamination by adventitious agents (viruses, bacteria, fungal agents, prions, etc.) requiring a control strategy to ensure their absence. Vaccines, immunological drugs that belong to the class of biological drugs, also have even more specificities (see “mRNA-based vaccines”), but it is especially the ATMPs that have additional requirements aimed at limiting the risks, thus for GTMP it is necessary to test the capacity of integration of the nucleic acid sequences in the genome, the functionality of these sequences, the risk of oncogenicity, etc. If viruses are used for the delivery of nucleic acids, the replication capacity of the viruses or microorganisms used *in vivo* must be tested. For sCTMP or if cells are used in GTMP, the origin of the cells (autologous, allogeneic, xenogeneic), the capacity to proliferate and/or differentiate and to induce an immune response, the level of cell manipulation, the combination of cells with bioactive molecules or structural materials, etc. must be controlled (35).

### The different RNA-based medicinal products and their statuses inside the European union

**FIGURE 2:**
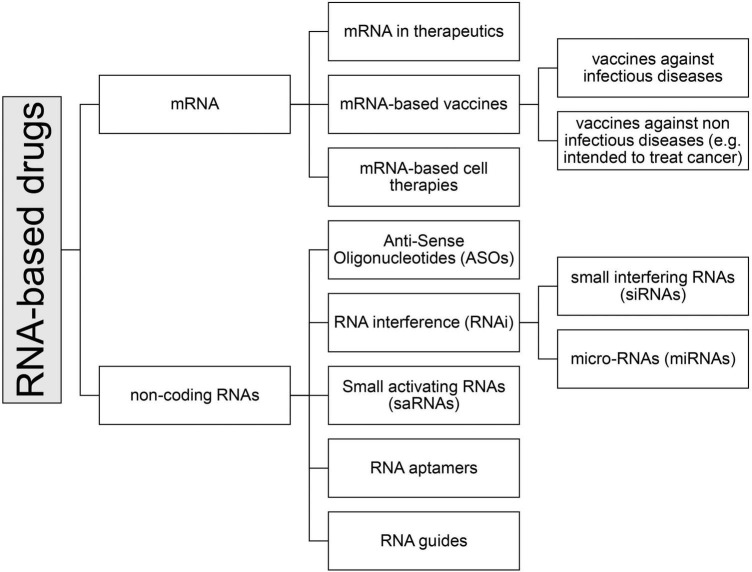
Classification of RNA-based drugs.

Among the medicinal products containing RNA, there are currently seven types of drugs that can be further subdivided into subcategories (see Figure 2):

• mRNA
• non-coding RNAs
  - Antisense oligonucleotides (ASOs)
  - Small interfering (si) and micro (mi)-RNA (RNA interference, iRNA)
  - Small activating RNA (saRNA)
  - RNA aptamers
  - RNA guides (gRNA)

Some of these drugs have already been approved and many are under development (Table 1).

For each type, we will discuss the legal status according to the EU legislation.

#### mRNA medicinal products

Among medicinal products containing mRNA, we can differentiate different subcategories:

• the general case for mRNA in therapeutics,
• 3 specific cases:
  ∘ mRNA-based vaccines,
  ∘ mRNA-based cell therapies as “therapeutic vaccines” against cancer,
  ∘ mRNA-based CAR-T cells.

##### General case for mRNA in therapeutics

The *Committee for Advanced Therapies* (*CAT;* This advisory committee, which reports to the EMA, is responsible for providing non-binding opinions on ATMPs, including their classification) has ruled that an mRNA meets the definition of a gene therapy medicinal product (GTMP) (36). In fact, several mRNAs have been classified by the CAT as GTMPs. Such mRNAs allow the synthesis of a protein such as the human glucose debranching enzyme, which is used in the treatment of glycogen storage disease III (GSD3) (18) or an antibody.

Indeed, these mRNAs are biological medicinal products as they are produced from a biological source, e.g., a DNA plasmid *in vitro* transcribed by recombinant enzymes. Moreover, the mRNA produced in this way is considered as a recombinant nucleic acid. This point will be further discussed paragraph “The classification of some RNA-based medicinal products in the GTMPs emphasizes the need for a definition of the term “recombinant” and an extension to other genetic techniques.” Finally, from the CAT’s point of view, the administration of mRNA corresponds to “the addition of a genetic sequence” (this is the justification given in the case of the drug for treating a glycogen storage disease) and its therapeutic action depends directly on the nucleic acid used.

##### mRNA-based vaccines

Although mRNA-based vaccines coding for pathogens or tumor antigens can be directly injected either to prevent or treat infectious diseases or to treat cancers, according to the EU, these two kinds of vaccines are not categorized under the same status. Indeed, there is a regulatory prohibition on including mRNA-based vaccines against infectious diseases, but not “cancer vaccines,” in the scope of GTMPs, making the term “cancer vaccines,” that is used in the scientific community (37–40), an invalid term when used in a regulatory context in the EU (31). In order to avoid misinterpretation, due to the frequent use of the term “cancer vaccine” in the literature, it is likely that the legislator intentionally used the wording “vaccines against infectious diseases” in the exclusionary phrase, otherwise the legislator would have used the term “vaccine” only.

The question of why vaccines against infectious diseases have been excluded is difficult to answer with certainty. Vaccines belong to the category of immunological medicinal products. The definition specifies that they are: “*agents used to produce active immunity, such as cholera vaccine, BCG, polio vaccines, smallpox vaccine”* (27). The vaccines listed in the definition are all vaccines intended to prevent an infectious disease. Although these are only examples, they do not include therapeutic mRNA-based “vaccines” against cancers. This could be explained by the fact that the definition has not changed since 1975, a period when there was no cancer “vaccine.” However, the exclusion text in the more recent GTMPs definition specifies “*vaccines against infectious diseases*” and not just “*vaccines*.” In the same spirit, the definition of vaccines given by the European pharmacopeia, provides that a vaccine produces active immunity in man against an infectious agent (32). But this one, which is revised very regularly, does not include cancer “vaccines” in its definition.

Another explanation suggested by Hinz (29) and by Nappi and Galli (31) is that vaccines against infectious diseases are prophylactic although “vaccines” against cancer are curative. While this could be an important justification, it should not be forgotten that HIV vaccines are therapeutic vaccines, especially as mRNA-based HIV vaccines are under development (41). Moreover, some vaccines have been excluded from the scope of ATMP by the CAT, because they act on infectious diseases, when they are not prophylactic but curative (42).

Two other explanations concerning public health could explain the special place of vaccines against infectious pathogens. The first relates to the target population: a very large healthy population, mostly including children. The second, which is a consequence of the first, is the specific regulation of vaccines, adapted to this mass use of a drug in a population. Let us mention the possibility, given by Article 114 of the consolidated Directive 2001/83/EC, for a Member State, in the interest of public health (“immunological medicinal products used in public health immunization programs”), to require the holder of an authorization for marketing to “*submit samples from each batch of the bulk and/or the medicinal product for examination by an Official Medicines Control Laboratory*” (OMCL). The competent authorities issue a “Batch Release Certificate” when the results are satisfactory. This is known as “Official Control Authority Batch Release” (OCABR).

In conclusion, it is clear that the specification “against infectious diseases” is especially important as vaccines that induce immunity to an infectious disease are excluded from GTMP scope, while mRNA-based “therapeutic vaccines” which are directly injected and induce immunity to a non-infectious disease will be considered as GTMP since they follow the criteria developed in paragraph a). This classification involves different constraints for development and marketing as only GTMP categorization requires specific tests specially to assess genome integration. This point will be discussed more generally for all RNA-based drugs in paragraph “The different RNA-based drug statuses between the EU and the USA need to be harmonized”.

##### mRNA-based cell therapies as “therapeutic vaccines” against cancer

Besides mRNA-based cancer “therapeutic vaccines” which are directly injected in patients, another approach consists in injecting autologous dendritic cells (DCs) which have been loaded *ex vivo* with *in vitro* transcribed mRNA coding tumor antigens.

Such a case was submitted to the CAT. In this instance it was an autologous dendritic cell immunotherapy consisting of autologous mature DCs co-electroporated with the *in vitro* transcribed autologous renal cell RNA and CD40L RNA. The EMA/CAT considered that the product falls within the definition of a sCTMP (43). According to the CAT, the product is considered not to comply with the complete requirements for a GTMP as the claim that mRNA in this case is administrated “with a view to adding a genetic sequence” is not fulfilled. The explanation is that the mRNA is probably no longer present at the time of the administration to the patient. Indeed, some authors, have hypothesized that because of the short half-life of mRNA in the modified cells, probably little or no residual mRNA will remain inside the cells administered to the patients. Indeed, in this particular case, the mRNA is used in the manufacturing process and is no longer part of the final product. The same would have been true if, instead of using mRNA, an antigenic protein had been used. So, for them, this can’t be considered as a GTMP (44). Moreover, the action of the drug depends not directly on the mRNA, but on the cells that have been modified. So, the product was categorized as a sCTMP as the injected cells had undergone a so-called substantial manipulation.

Of note, if the mRNA had not been a non-replicating mRNA, but a self-replicating (self-amplifying) mRNA construct, the half-life of RNA would have been far longer (45), resulting in the presence of mRNA in the electroporated cells at the time of injection. So, in this case, still hypothetical to our knowledge, the drug would be classified as a GTMP.

##### Specific case of mRNA-encoded chimeric antigen receptor

Another immunotherapy approach in cancer is based on the redirection of T cells against tumor cells by stable integration of a chimeric antigen receptor (CAR) which recognizes tumor antigens independently of MHC. Current CAR-T cells, which are genetically modified by adding DNA *ex vivo*, are classified as GTMP. The previous decision concerning DC has strong implications since there are CAR-T cells under development that are modified using *ex vivo* mRNA electroporation (7, 8). As seen above, we should consider that they should be classified as sCTMP.

Moreover, a new strategy is under development to generate transient CAR-T cells *in vivo* using mRNA lipid nanoparticles (9). In this case of *in vivo* transcribed RNA, the status would not be a sCTMP, but GTMP. Indeed, here, the mRNA is directly injected to the patient and in such a case, the administration of mRNA corresponds to “the addition of a genetic sequence” and its therapeutic action depends directly on the nucleic acid used.

Thus, depending on the methodology, *ex vivo* electroporated T cells with CAR mRNA or *in vivo* targeting of T cells with CAR mRNA, the drug can be either a sCTMP or a GTMP. This difference in status, which at first glance may seem surprising and inconsistent, is justified. Indeed, the controls to be carried out are very different; the production, the logistics inherent in the latter and even the administration of cell-based drugs involve very different controls compared to the production of drugs based on mRNA encapsulated in lipid nanoparticles (LNPs). Moreover, the controls exerted before administration cannot be the same: for example, while it is easy to control the protein translation of mRNA in a cell manipulated *ex vivo* before its administration to the patient, this is very complex if the mRNA is injected *in vivo* through LNPs. In the latter case, it is very difficult to know if the translation was efficient and did not generate errors.

#### Non-coding RNA: Antisense oligonucleotides, small interfering RNAs, micro-RNAs and small activating RNAs (46–48)

Antisense oligonucleotides are short nc single-stranded (ss) RNA sequences that modulate gene expression by binding to pre-mRNAs or mRNAs and acting by either occupancy-induced (pre)-mRNA RNase degradation or by occupancy-only mechanisms (steric blocking). They can also modulate RNA splicing to produce functional or preferred genetic products.

Both small interfering RNAs (siRNAs) and microRNAs (miRNAs) which mimic natural miRNAs are short nc double-stranded RNAs that modulate mRNA expression in the cytoplasm. They recognize target transcripts via the RNA-induced silencing complex (RISC) leading to translation inhibition or mRNA cleavage. The development of miRNAs is less advanced than that of siRNAs.

Small activating RNAs (saRNA) are nc short double-stranded oligonucleotides that have the same structure as siRNAs, however, their biological function is totally different (47, 49). Indeed, saRNAs act in the nucleus, induce transcription and increase gene expression by targeting gene promoters (47, 50).

As far as we know, ASOs, siRNA, miRNA and saRNA are currently chemically synthesized. Thus, they are not biological medicinal products, and they can’t by classified in ATMPs. They can only pretend to the status of “chemical” (“simple”) medicinal products.

However, bioengineered si- or miRNA (biological-bioengineered RNA agents called BERAs) aiming at better mimicking physiological si- or mi-RNA molecules (46, 51) are in development. These BERAs seem to better capture the structure, function, and safety properties of natural RNAs (52), probably avoiding the toxicity induced by excessively chemically modified RNA and/or the lack of necessary posttranscriptional modifications occurring in natural RNAs (53). The development of this technology raises the question of the categorization of drugs having the same mechanism of action but being produced chemically or biologically.

#### RNA aptamers

RNA aptamers bind to and inhibit a wide variety of targets (including proteins, peptides, DNAs, RNAs) by virtue of their tertiary structure. They are produced by chemical synthesis and thus categorized as simple medicinal products (46, 51).

#### RNA guides/CRISPR Cas9 nuclease

The CRISPR/Cas9-based genome editing and therapy based on exogenous guide RNA and foreign Cas nuclease CRISPR-Cas9 is revolutionizing gene therapy. CRISPR-Cas9 is a complex, two-component system using a short guide RNA (gRNA) sequence to direct the Cas9 endonuclease to target a gene. While the guide RNA is produced mainly by chemical synthesis, it is the nuclease that determines the status of the drug, i.e., either a biological medicinal product when it is injected as a recombinant protein or a GTMP when it is produced by *in vivo* injection of mRNA (54).

**FIGURE 3:**
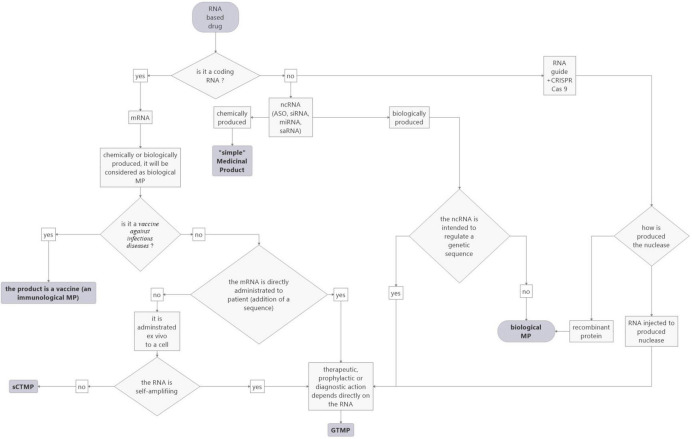
Proposed flowchart to facilitate the categorization of RNA-based medicinal products according to the current EU rules.

In conclusion, RNA-based drugs are categorized in different statuses depending on their targets (vaccines against infectious disease), whether they are loaded *ex vivo* (CAR-T cells, dendritic cells), or for ncRNAs, on their production (chemical or biological). Concerning the latter, the evolution of technology makes things more complex, as similar RNA drugs may be classified in different statuses. This raises the question of the relevance of controls for development and marketing. In order to help to categorize RNA-based drugs according to the current rules, we propose a flowchart (see Figure 3).

## Critical issues in the categorization of RNA-based medicinal products: Actionable recommendations

### Issues or questions when confronting RNA-based medicinal products with the definition of gene therapy medicinal products

#### RNA-based medicinal products question the relevance of maintaining the current European union definition of a biological medicinal product

In theory, a drug containing a chemically synthesized RNA cannot claim the status of a biological drug, and therefore *in extension of* a vaccine or a GTMP. In the case where some RNA could be both chemically and biologically synthesized, this could lead to having two medicinal products with the same indication and the same composition (with the only difference being the source of the RNA), but which would not have the same status at all. This would be problematic and cruelly lacking in consistency. Thus, CAT explained: “*long chain mRNAs cannot yet be produced via chemical synthesis. However, when this becomes possible, the regulatory status of such synthetic RNAs needs to be considered, as it should be avoided to have similar products being covered by different legal frameworks*” (36).

In order to overcome this problem, the EMA, like the European Commission, considers that “RNA derived products should be considered as biologicals, even if not derived from a biological source” (55). This consideration was given in the context of COVID-19 vaccines, and it can only concern *a priori* mRNA technology. Consequently, whatever the mode of production of mRNA, biosynthesis or chemical synthesis, RNA, given its nature and properties, could be considered a biological substance. The CAT is aware that this position should be discussed since it “would extend the ATMP definition also to synthetic RNAs (such as small interfering RNAs) or synthetic DNA oligonucleotides” (55).

Of note, as indicated above, new approaches to produce bio-engineered si- or mi-RNAs are already a reality (see BERA, paragraph “Non-coding (nc) RNA: ASOs, siRNA, miRNA and saRNA”). They will probably lead to a new class of iRNA molecules, similar to chemical iRNAs but which should be categorized as biological medicinal products and thus possibly as GTMPs. Therefore, according to CAT, considering RNA as a biological substance could prevent categorizing similar RNA drugs in very different statuses, depending on their mode of production. But one should be aware that this change in classification would not be without consequence since, if GTMP status is retained, these RNA-based drugs would then have to follow the very specific GTMP regulations (see below).

This type of problem highlights the possible inadequacy of the European definition of a biological medicinal product. Indeed, it seems that the current definition of a biological medicinal product is no longer consistent with the scientific reality. Some products would deserve to be placed under the status of a biological medicinal product so that they can undergo the appropriate controls to ensure the protection and safety of patients.

A modification of the definition of a biological medicinal product, which is fundamental because it conditions its potential categorization in the GTMP status, so that it is no longer only based on its biological source but possibly on its control, would resolve this issue and avoid complex situations where the EMA and the European Commission have to issue numerous *ad hoc* rules, creating a sort of regulatory patch, to fit this or that product into the existing regulatory categories.

Thus, a biological medicinal product should be a medicinal product whose active substance:

• is produced or extracted from a biological source

**and/or**

• needs for its characterization and the determination of its quality a combination of physico-chemical-biological testing, together with the production process and its control.

#### The classification of some RNA-based medicinal products in the gene therapy medicinal products emphasizes the need for a definition of the term “recombinant” and an extension to other genetic techniques

There is no legal definition of recombinant nucleic acid. According to the spirit of the Regulation (EC) N*^o^* 1394/2007 and some CAT recommendations, it seems that the word “recombinant” should be understood as an artificial recombination of nucleic acids. For example, a chimeric adenovirus obtained “simply” by a process of bio-selection is not an ATMP since it was not manipulated and therefore the therapeutic action is not mediated by any recombinant nucleic acid (56).

We can also refer to the definition of “recombinant techniques” in Directive 2001/18/EC on the deliberate release into the environment of genetically modified organisms: “*recombinant nucleic acid techniques involving the formation of new combinations of genetic material by the insertion of nucleic acid molecules produced by whatever means outside an organism, into any virus, bacterial plasmid or other vector system and their incorporation into a host organism in which they do not naturally occur but in which they are capable of continued propagation*.”

Of note is that the US definition of recombinant nucleic acid is: “molecules that (a) are constructed by joining nucleic acid molecules and (b) that can replicate in a living cell.”

Currently, the CAT considers that messenger RNAs (mRNA) that are produced biosynthetically (transcribed *in vitro* from a DNA template) fulfill the definition of a GTMP, and so are considered as recombinant whatever they are self-replicating or not.

So, it seems that a nucleic acid is said to be recombinant if it is the result of a laboratory assembly of nucleic acids. Currently, in the case of mRNA drugs, it is safe to say that they should be recombinant, because of the way they are produced. Indeed, mRNAs produced by *in vitro transcription* are most generated using a plasmid DNA template. The question is if the DNA template is PCR products or synthetic oligonucleotides.

This could be solved by modifying the definition to include synthetic nucleic acids.

### The different RNA-based drug statuses between the European union and the United States need to be harmonized

For both the EU and the USA, the biological origin of the drug matters, so ASOs, siRNA, miRNA, saRNA and aptamers, which are chemically produced, are considered as “chemical drugs.”

The problem is rather with mRNA because of the differences in the definitions of GTMP between the EU and the USA.

According to FDA guidance, gene therapy is defined in the United States as: “*a medical intervention based on modification of the genetic material of living cells. Cells may be modified ex vivo for subsequent administration to humans or may be altered in vivo by gene therapy given directly to the subject. When the genetic manipulation is performed ex vivo on cells which are then administered to the patient, this is also a form of somatic cell therapy. The genetic manipulation may be intended to have a therapeutic or prophylactic effect or may provide a way of marking cells for later identification. Recombinant DNA materials used to transfer genetic material for such therapy are considered components of gene therapy and as such are subject to regulatory oversight*” (57).

The US definition only evokes the possibility of using recombinant DNA, whereas the European definition evokes the use of recombinant nucleic acid (which can therefore refer to both DNA and RNA). So, in the US, mRNA technologies should not be considered as gene therapy.

This divergence results most likely from a different vision of gene therapy. In fact, the FDA relies mainly on the mechanism of action to define gene therapy, which requires an interaction with human DNA in the nucleus (58). Conversely, the EMA seems to focus on the composition of the drug (recombinant nucleic acid), and does not take into account the modification of DNA in the nucleus or even the durability of the modification over time (59).

So, such differences resulting from different visions of what mRNA and gene therapies are, lead to discrepancies in the status of products marketed on either side of the Atlantic. Indeed, as seen above, an mRNA-based drug will be a GTMP in Europe (except for a vaccine against infectious disease) while it will not be so in the USA (of note in the USA, mRNA vaccines against infectious diseases are also categorized as vaccines).

This divergence has consequences in terms of requirements for the Marketing Authorization to ensure the safety and security for patients (see below).

The solution could come from the ICH which is in charge of harmonizing the MA files and requirements between its members. A draft of the ICH guideline S12 gives a definition of gene therapy that is quite far from the European and US definitions (60).

The ICH, in its S12 guideline (still in draft form at this time) has introduced a singular definition of medicinal products for gene therapy: “*Gene therapy (GT) products within the scope of this guideline include products that mediate their effect by the expression (transcription or translation) of transferred genetic materials. Some examples of GT products can include purified nucleic acid (e.g., plasmids and RNA), microorganisms (e.g., viruses, bacteria, fungi) genetically modified to express transgenes (including products that edit the host genome), and ex vivo genetically modified human cells. Products that are intended to alter the host cell genome in vivo without specific transcription or translation (i.e., delivery of a nuclease and guide RNA by non-viral methods) are also covered in this guidance.*”

So, this definition could resolve the question of the heterogeneity of similar RNA-based drugs being produced by a chemical or biological approach as the mode of production is not mentioned neither the question of recombinant nucleic acids. However, ncRNAs which do not mediate their effect by the expression (transcription or translation) of transferred genetic materials and do not alter the host cell genome are still not considered gene therapy whereas, according to the EU definition, they could be in the case where they are biologically produced and recombinant (BERAs).

Concerning *ex vivo* genetically human modified cells, they are also considered gene therapy in the ICH definition. This could resolve the question of the different statuses for CAR-T cells depending on the strategy used to prepare them (see paragraph “Specific case of mRNA-encoded Chimeric Antigen Receptor”).

Finally, direct in-body gene editing using CRISPR/Cas9, fall within the scope of the proposed ICH regulation whatever, once again, is the approach. Whereas according to EMA/CAT nanoparticles containing the gene editing components (CRISPR/Cas9 and the single guide RNAs) intended for the treatment of Recessive Dystrophic epidermolysis bullosa, do not fall within the definition of ATMP as the product does not contain an active substance which contains a recombinant nucleic acid administered to human beings with a view to regulating, repairing, adding, deleting a genetic sequence (18).

**FIGURE 4:**
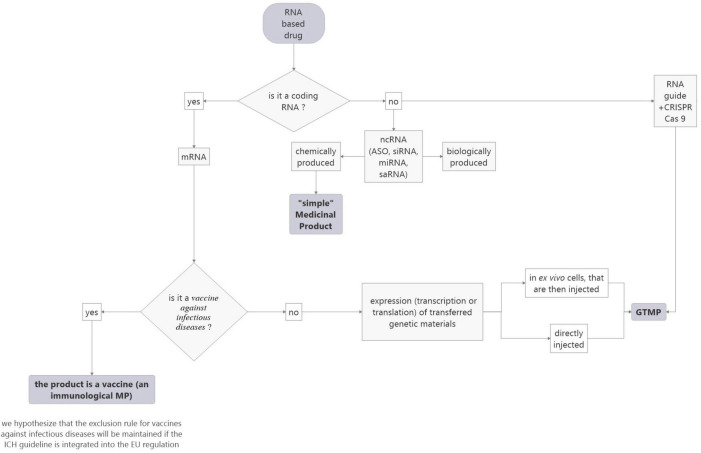
Proposed flowchart to facilitate the categorization of RNA-based medicinal products according to the ICH rules project.

In conclusion, this definition simplifies the categorization of mRNA-based drugs compared to the EU regulation and allows harmonizing the EU and US regulations, as it does not consider the origin of RNA. It also solves the question of genetically modified cells and of genome-editing methods (see Figure 4). However, it does not include nc RNA-based drugs which can be produced chemically or biologically. This point deserves to be addressed.

## Discussion

According to the EU legislation, RNA-based drugs fall under several different statuses depending, for vaccines, on their targets – infectious *vs*. non-infectious diseases – and for non-vaccine drugs, on the type of RNA substance and its production. For vaccines, although subject to discussion, the target criterion is easy to use for classification. Moreover, it is harmonized with the US legislation. On the contrary, for non-vaccine RNA-based drugs, the current evolution of technology in this field and the possibility to produce similar drugs using different approaches, make it necessary to amend the legislation to avoid similar products being covered by different legal frameworks. In particular, a clarification of the definition of a biological medicinal product considering not only the biological source of the product but its physico-chemical-biological control, and of a recombinant nucleic acid, could solve some problems. However, such modifications, in addition to not solving all the problems, do not address the issue of the international harmonization which is necessary. Indeed, there are currently major differences between the EU and the US legislations. In this respect, the proposed definition of a gene therapy product by the ICH represents an important step forward. The question of nc RNA-based drugs which are not concerned by this proposition should, however, be discussed with regard to the most important criterion to consider for a gene therapy drug, i.e., safety and protection of patients. According to the EU, GTMPs require specific tests or trials to evaluate the risk of germ-line transmission, insertional mutagenesis, tumorigenicity, and embryo/fetal and perinatal toxicity and, according to article 15 of Regulation 1394/2007 (28), GTMPs have an obligation to provide safety and efficacy data for 30 years after the expiry date of the drug, what is beyond the requirements of the classical pharmacovigilance.

In the case of RNA-based drugs, the question is that of the relevance of these stringent tests to assess the consequences of a possible integration of the administrated RNA in the cell genome following its retro-transcription into cDNA. Retro-transcription of RNA, independently of a retroviral retro-transcriptase (RT), may involve retrotransposons LINE-1 (long interspersed element-1), which are remnants of ancient retroviral infections containing endogenous RT; they represent about 17% of the human genome, among them few copies are active. These elements predominantly transpose their own copies; however, they can also promote the retrotransposition of other cellular RNAs, including short interspersed elements (SINEs) and less commonly, small nuclear non-coding RNAs and mRNA (61–66). Although this possibility is considered rare and the risk for an RNA-based drug to integrate has not yet been reported, it must be noticed that higher level of activity of transposable elements has been reported in some diseases, especially in cancer (66) or in viral infection (67). Thus, in the current state of knowledge and given the high potential of development of RNA-based drugs, this scientific issue should be considered from a regulatory point of view and confronted with a possible application of the precautionary principle that enables decision-makers to adopt precautionary measures when scientific evidence about human health hazard is uncertain and the stakes are high (68). Therefore, RNA-based drugs which are not categorized GTMPs such as mRNA vaccines and ncRNA should at least be subject to specific guidelines.

Thus, while the international harmonization proposed by the ICH seems inevitable and desirable, it will not solve all the problems of categorization. The forthcoming revision of European regulations, which should incorporate the ICH definition, could lead to a greater clarity in categorization, but vigilance will be required, since a minor change in the regulations could have unexpected cascading repercussions on future and past categorizations. The notion of *primum non-nocere* should therefore be applied in regulatory matters.

One solution could come from drafting specific guidelines for RNA drugs. These guidelines could go beyond the differences in categorization and thus be applied across the board. Logically, they should make it possible to establish specific safety rules, particularly if two identical but differently produced RNAs are placed on the market.

In addition to the guidelines already applicable according to the legal categorization, such guidelines could also ensure the safest possible development, by integrating the appropriate toxicological studies according to the risk of oncogenesis, integration, cell migration, etc.

As far as production is concerned, the guidelines in force provide numerous guarantees, and the notion of risk-based approach (for ATMPs) allows for the closest possible adaptation to each medicinal product profile. However, specific guidelines for verifying the sequence produced and the three-dimensional conformation could perhaps prove useful.

Finally, specific guidelines for vigilance could be interesting, in particular to ensure long-term monitoring. The reinforcement of the follow-up of chemically produced RNAs should be considered.

## Conclusion

The regulation of EU RNA-based medicinal products is very complex, and this is likely to become more pronounced with the evolution of technology. Moreover, there are profound disparities between the EU and the US classifications.

The current EU regulation raises the question of a biological medicinal product which should be modified to consider not only the biological source of the product but its physico-chemical-biological control to include all RNA-based drugs whatever their production.

Considering RNA-based drugs and gene therapy, the ICH definition proposal should allow simplification and harmonization between the EU and US regulations for mRNA-based drugs, among them mRNA-based cell therapies as well as for gene-editing therapies and facilitate marketing. However, it will not solve all the issues related to RNA-based drugs, especially since some of them are not covered by the definition, such as mRNA vaccines and ncRNA. Therefore, the drafting of specific guidelines could ensure patient safety by imposing specific controls.

## Author contributions

All authors listed have made a substantial, direct, and intellectual contribution to the work, and approved it for publication.

## References

[1] European Medicines Agency. *Comirnaty.* (2020). Available online at: https://www.ema.europa.eu/en/medicines/human/EPAR/comirnaty (accessed Sept 1, 2022).

[2] European Medicines Agency. *Spikevax.* (2021). Available online at: https://www.ema.europa.eu/en/medicines/human/EPAR/spikevax (accessed Sept 1, 2022).

[3] NIH. *Safety, Tolerability, and Pharmacokinetics of UX053 in Patients With Glycogen Storage Disease Type III (GSD III).* (2021). Available online at: https://clinicaltrials.gov/ct2/show/NCT04990388?cond=GSD3&draw=2&rank=1 (accessed April 7, 2022).

[4] Sahin U Derhovanessian E Miller M Kloke B-P Simon P Löwer M Personalized RNA mutanome vaccines mobilize poly-specific therapeutic immunity against cancer. *Nature.* (2017) 547:222–6. 10.1038/nature23003 28678784

[5] Miller M Sahin U Derhovanessian E Kloke BP Simon P Bukur V IVAC MUTANOME: a first-in-human phase I clinical trial targeting individual mutant neoantigens for the treatment of melanoma. *Ann Oncol.* (2017) 28:xi1–2.

[6] Amin A Dudek AZ Logan TF Lance RS Holzbeierlein JM Knox JJ Survival with AGS-003, an autologous dendritic cell-based immunotherapy, in combination with sunitinib in unfavorable risk patients with advanced renal cell carcinoma (RCC): phase 2 study results. *J Immunother Cancer.* (2015) 3:14. 10.1186/s40425-015-0055-3 25901286 PMC4404644

[7] SparkCures. *Descartes-08 (SparkCures Myeloma Clin. Trials SparkCures).* (2020). Available online at: https://sparkcures.com/treatment/321/descartes-08 (accessed Dec 15, 2021).

[8] NIH. *Descartes-08 Consolidation Treatment in Patients With High-Risk Multiple Myeloma Who Have Residual Disease After Induction Therapy.* (2021). Available online at: https://clinicaltrials.gov/ct2/show/NCT04816526?term=Descartes-08&draw=2&rank=1 (accessed Dec 15, 2021).

[9] Rurik JG Tombacz I Yadegari A Mendez Fernandez PO Shewale SV Li L CAR T cells produced in vivo to treat cardiac injury. *Science.* (2022) 375:91–6. 10.1126/science.abm0594 34990237 PMC9983611

[10] NIH. *Efficacy, Safety, and Tolerability of Remlarsen (MRG-201) Following Intradermal Injection in Subjects With a History of Keloids.* (2018). Available online at: https://clinicaltrials.gov/ct2/show/NCT03601052 (accessed April 7, 2022).

[11] Gallant-Behm CL Piper J Lynch JM Seto AG Hong SJ Mustoe TA A microRNA-29 mimic (Remlarsen) represses extracellular matrix expression and fibroplasia in the skin. *J Invest Dermatol.* (2019) 139:1073–81. 10.1016/j.jid.2018.11.007 30472058

[12] NIH. *Study of Lademirsen (SAR339375) in Patients With Alport Syndrome (HERA).* (2016). Available online at: https://clinicaltrials.gov/ct2/show/NCT02855268 (accessed April 7, 2022).

[13] Setten RL Lightfoot HL Habib NA Rossi JJ. Development of MTL-CEBPA: small activating RNA drug for hepatocellular carcinoma. *Curr Pharm Biotechnol.* (2018) 19:611–21. 10.2174/1389201019666180611093428 29886828 PMC6204661

[14] NIH. *Phase I Study of RNA Oligonucleotide, MTL-CEBPA, Atezolizumab and Bevacizumab in Patients With Advanced Hepatocellular Carcinoma.* (2021). Available online at: https://clinicaltrials.gov/ct2/show/NCT05097911?cond=mtl-cebpa&draw=2&rank=1 (accessed April 7, 2022).

[15] NIH. *Radomised Phase II Study of MTL-CEBPA Plus Sorafenib or Sorafenib Alone (OUTREACH2).* (2021). Available online at: https://clinicaltrials.gov/ct2/show/NCT04710641?cond=mtl-cebpa&draw=2&rank=2 (accessed April 7, 2022).

[16] NIH. *Study to Evaluate Safety, Tolerability, Pharmacokinetics, and Pharmacodynamics of NTLA-2001 in Patients With Hereditary Transthyretin Amyloidosis With Polyneuropathy (ATTRv-PN) and Patients With Transthyretin Amyloidosis-Related Cardiomyopathy (ATTR-CM).* (2020). Available online at: https://clinicaltrials.gov/ct2/show/NCT04601051 (accessed April 7, 2022).

[17] Gillmore JD Gane E Taubel J Kao J Fontana M Maitland ML CRISPR-Cas9 in vivo gene editing for transthyretin amyloidosis. *N Engl J Med.* (2021) 385:493–502. 10.1056/NEJMoa2107454 34215024

[18] European Medicines Agency. *Scientific Recommendation on Classification of Advanced Therapy Medicinal Products. EMA/140033/2021.* Amsterdam: European Medicines Agency (2021).

[19] European Medicines Agency. *Spinraza.* (2018). Available online at: https://www.ema.europa.eu/en/medicines/human/EPAR/spinraza (accessed Sept 1, 2022).

[20] European Medicines Agency. *Tegsedi.* (2018). Available online at: https://www.ema.europa.eu/en/medicines/human/EPAR/tegsedi (accessed Sept 1, 2022).

[21] European Medicines Agency. *Waylivra.* (2018). Available online at: https://www.ema.europa.eu/en/medicines/human/EPAR/waylivra (accessed Sept 1, 2022).

[22] European Medicines Agency. *Onpattro.* (2018). Available online at: https://www.ema.europa.eu/en/medicines/human/EPAR/onpattro (accessed Sept 1, 2022).

[23] European Medicines Agency. *Givlaari.* (2020). Available online at: https://www.ema.europa.eu/en/medicines/human/EPAR/givlaari (accessed Sept 1, 2022).

[24] European Medicines Agency. *Oxlumo.* (2020). Available online at: https://www.ema.europa.eu/en/medicines/human/EPAR/oxlumo (accessed Sept 1, 2022).

[25] European Medicines Agency. *Leqvio.* (2020). Available online at: https://www.ema.europa.eu/en/medicines/human/EPAR/leqvio (accessed Sept 1, 2022).

[26] European Medicines Agency. *Reflection Paper on Classification of Advanced Therapy Medicinal Products EMA/CAT/600280/2010 Rev.1.* Amsterdam: European Medicines Agency (2015).

[27] European Union. *Directive 2001/83/EC of the European Parliament and of the Council of 6 November 2001 on the Community Code Relating to Medicinal Products for Human Use.* Maastricht: European Union (2001).

[28] European Union. *Regulation (EC) No 1394/2007 of the European Parliament and of the Council of 13 November 2007 on Advanced Therapy Medicinal Products and Amending Directive 2001/83/EC and Regulation (EC) No 726/2004.* Maastricht: European Union (2007).

[29] Hinz T Kallen K Britten CM Flamion B Granzer U Hoos A *The European Regulatory Environment of RNA-Based Vaccines. RNA Vaccines: Methods and Protocols.* New York: Springer (2017). p. 203–22. 10.1007/978-1-4939-6481-9_13 27987152

[30] Sahin U Kariko K Tureci O. mRNA-based therapeutics-developing a new class of drugs. *Nat Rev Drug Discov.* (2014) 13:759–80. 10.1038/nrd4278 25233993

[31] Nappi F Galli MC. Chapter 4 – Regulatory aspects of cancer immunotherapy in Europe. In: Buonaguro L Van Der Burg S editors. *Cancer Vaccines as Immunotherapy of Cancer.* Cambridge, MA: Academic Press (2022). p. 75–84. 10.1016/B978-0-12-823901-8.00009-1

[32] European Directorate for the Quality of Medicines and HealthCare. Vaccine for human use 04/2022:0153. 10th ed. In: European Pharmacopoeia, editor. *Council of Europe.* Strasbourg: European Directorate for the Quality of Medicines and HealthCare (2019).

[33] Berkowitz SA Engen JR Mazzeo JR Jones GB. Analytical tools for characterizing biopharmaceuticals and the implications for biosimilars. *Nat Rev Drug Discov.* (2012) 11:527–40. 10.1038/nrd3746 22743980 PMC3714370

[34] European Medicines Agency. *Biosimilars in the EU: Information Guide for Healthcare Professionals.* Amsterdam: European Medicines Agency (2019).

[35] Guerriaud M. Les médicaments de thérapie innovante – Statut juridique. In: LexisNexis editor. *Juris Classeur Droit Pharmaceutique Fascicule*. Paris: LexisNexis (2022). p. 61–70.

[36] European Medicines Agency. *Committee for Advanced Therapies. Minutes of the Meeting on 02-04 December 2020. EMA/CAT/162004/2021.* Amsterdam: European Medicines Agency (2021).

[37] Sahin U Türeci Ö. Personalized vaccines for cancer immunotherapy. *Science.* (2018) 359:1355–60. 10.1126/science.aar7112 29567706

[38] Vermaelen K. Vaccine strategies to improve anti-cancer cellular immune responses. *Front Immunol.* (2019) 10:8. 10.3389/fimmu.2019.00008 30723469 PMC6349827

[39] Morse MA Gwin WR III Mitchell DA. Vaccine therapies for cancer: then and now. *Target Oncol.* (2021) 16:121–52. doi10.1007/s11523-020-00788-w 33512679 10.1007/s11523-020-00788-w PMC7845582

[40] Wu M Wang S Chen JY Zhou LJ Guo ZW Li YH. Therapeutic cancer vaccine therapy for acute myeloid leukemia. *Immunotherapy.* (2021) 13:863–77. 10.2217/imt-2020-0277 33955237

[41] Zhang P Narayanan E Liu Q Tsybovsky Y Boswell K Ding S A multiclade env-gag VLP mRNA vaccine elicits tier-2 HIV-1-neutralizing antibodies and reduces the risk of heterologous SHIV infection in macaques. *Nat Med.* (2021) 27:2234–45. 10.1038/s41591-021-01574-5 34887575

[42] European Medicines Agency. *Scientific Recommendation on Classification of Advanced Therapy Medicinal Products. Live Recombinant Lentiviral Vectors Encoding HIV Epitopes to be Used for Therapeutic HIV Vaccination of HIV-1 Infected Patients.* Amsterdam: European Medicines Agency (2011).

[43] European Medicines Agency. *Scientific Recommendation on Classification of Advanced Therapy Medicinal Products. EMA/921464/2011.* Amsterdam: European Medicines Agency (2011).

[44] López-Paniagua M de la Mata A Galindo S Blázquez F Calonge M Nieto-Miguel T. Advanced therapy medicinal products for the eye: definitions and regulatory framework. *Pharmaceutics.* (2021) 13:347. 10.3390/pharmaceutics13030347 33800934 PMC8000705

[45] Kowalzik F Schreiner D Jensen C Teschner D Gehring S Zepp F. mRNA-based vaccines. *Vaccines.* (2021) 9:390. 10.3390/vaccines9040390 33921028 PMC8103517

[46] Yu A-M Choi YH Tu M-J. RNA drugs and RNA targets for small molecules: principles, progress, and challenges. *Pharmacol Rev.* (2020) 72:862–98. 10.1124/pr.120.019554 32929000 PMC7495341

[47] Moumné L Marie A-C Crouvezier N. Oligonucleotide therapeutics: from discovery and development to patentability. *Pharmaceutics.* (2022) 14:260. 10.3390/pharmaceutics14020260 35213992 PMC8876811

[48] Crooke ST Witztum JL Bennett CF Baker BF. RNA-targeted therapeutics. *Cell Metab.* (2018) 27:714–39. 10.1016/j.cmet.2018.03.004 29617640

[49] Voutila J Reebye V Roberts TC Protopapa P Andrikakou P Blakey DC Development and mechanism of small activating RNA targeting CEBPA, a novel therapeutic in clinical trials for liver cancer. *Mol Ther.* (2017) 25:2705–14. 10.1016/j.ymthe.2017.07.018 28882451 PMC5768526

[50] Kwok A Raulf N Habib N. Developing small activating RNA as a therapeutic: current challenges and promises. *Therap Deliv.* (2019) 10:151–64. 10.4155/tde-2018-0061 30909853

[51] Yu A-M Jian C Yu AH Tu M-J. RNA therapy: are we using the right molecules? *Pharmacol Ther.* (2019) 196:91–104. 10.1016/j.pharmthera.2018.11.011 30521885 PMC6450780

[52] Ho PY Yu AM. Bioengineering of noncoding RNAs for research agents and therapeutics. *Wiley Interdiscip Rev RNA.* (2016) 7:186–97. 10.1002/wrna.1324 26763749 PMC4769674

[53] Duan Z Yu AM. Bioengineered non-coding RNA agent (BERA) in action. *Bioengineered.* (2016) 7:411–7. 10.1080/21655979.2016.1207011 27415469 PMC5094625

[54] Allen D Rosenberg M Hendel A. Using synthetically engineered guide RNAs to enhance CRISPR genome editing systems in mammalian cells. *Front Genome.* (2021) 2:617910. 10.3389/fgeed.2020.617910 34713240 PMC8525374

[55] European Medicines Agency. *Committee for Advanced Therapies. Minutes of the Meeting on 15-17 July 2020. EMA/CAT/510852/2020.* Amsterdam: European Medicines Agency (2020).

[56] European Medicines Agency. *Scientific Recommendation on Classification of Advanced Therapy Medicinal Products. EMA/348841/2012.* Amsterdam: European Medicines Agency (2012).

[57] Food and Drug Administration. *Guidance for Industry: Guidance for Human Somatic Cell Therapy and Gene Therapy.* Silver Spring, MD: Food and Drug Administration (1998).

[58] Food and Drug Administration. *Personal Communication: Questions About RNAm Drugs.* Silver Spring, MD: Food and Drug Administration (2022).

[59] Celis P. *Personal Communication: mRNA Drugs Regulation and Categorization. ATMP Office of EMA.* Amsterdam: European Medicines Agency (2022).

[60] European Medicines Agency. *International Council for Harmonisation of Technical Requirements for Pharmaceuticals for Human Use. Draft: Nonclinical Biodistribution Considerations for Gene Therapy Products s12.* Amsterdam: European Medicines Agency (2021).

[61] Esnault C Maestre J Heidmann T. Human LINE retrotransposons generate processed pseudogenes. *Nat Genet.* (2000) 24:363–7. 10.1038/74184 10742098

[62] Buzdin A Gogvadze E Kovalskaya E Volchkov P Ustyugova S Illarionova A The human genome contains many types of chimeric retrogenes generated through in vivo RNA recombination. *Nucleic Acids Res.* (2003) 31:4385–90. 10.1093/nar/gkg496 12888497 PMC169886

[63] Dewannieux M Esnault C Heidmann T. LINE-mediated retrotransposition of marked Alu sequences. *Nat Genet.* (2003) 35:41–8. 10.1038/ng1223 12897783

[64] Mandal PK Ewing AD Hancks DC Kazazian HH Jr. Enrichment of processed pseudogene transcripts in L1-ribonucleoprotein particles. *Hum Mol Genet.* (2013) 22:3730–48. 10.1093/hmg/ddt225 23696454 PMC3749862

[65] Moldovan JB Wang Y Shuman S Mills RE Moran JV. RNA ligation precedes the retrotransposition of U6/LINE-1 chimeric RNA. *Proc Natl Acad Sci U.S.A.* (2019) 116:20612–22. 10.1073/pnas.1805404116 31548405 PMC6789731

[66] Burns KH. Transposable elements in cancer. *Nat Rev Cancer.* (2017) 17: 415–24. 28642606 10.1038/nrc.2017.35

[67] Macchietto MG Langlois RA Shen SS. Virus-induced transposable element expression up-regulation in human and mouse host cells. *Life Sci Alliance.* (2020) 3:e201900536. 10.26508/lsa.201900536 31964680 PMC6977392

[68] Bourguignon D. *European Parliament, Directorate-General for Parliamentary Research Services. The Precautionary Principle: Definitions, Applications and Governance: In-depth Analysis: Publications Office.* Strasbourg: European Parliament (2016).

[69] European Medicines Agency. *Kyndrisa: Withdrawn Application.* (2018). Available online at: https://www.ema.europa.eu/en/medicines/human/withdrawn-applications/kyndrisa (accessed Sept 1, 2022).

[70] European Medicines Agency. *Macugen: Withdrawn Application.* (2018). Available online at: https://www.ema.europa.eu/en/medicines/human/withdrawn-applications/macugen (accessed Sept 1, 2022).

